# *Lactobacillus plantarum* Inoculants Delay Spoilage of High Moisture Alfalfa Silages by Regulating Bacterial Community Composition

**DOI:** 10.3389/fmicb.2020.01989

**Published:** 2020-08-12

**Authors:** Fengyuan Yang, Yanping Wang, Shanshan Zhao, Yuan Wang

**Affiliations:** ^1^Henan Provincial Key Laboratory of Ion Beam Bioengineering, School of Agricultural Science, Zhengzhou University, Zhengzhou, China; ^2^Henan Provincial Key Laboratory of Ion Beam Bioengineering, College of Physics, Zhengzhou University, Zhengzhou, China

**Keywords:** *Lactobacillus plantarum*, spoilage microorganism, alfalfa silage, adverse ensiling condition, high-throughput sequencing

## Abstract

The objective of this study was to investigate the mechanism of *Lactobacillus plantarum* (*L. plantarum*) involved in improving fermentation quality of naturally ensiled alfalfa under poor conditions. High-moisture wilted alfalfa was ensiled without inoculants (CK) or with inoculation of two *L. plantarum* additives (LPI and LPII). The pH and fermentation products of silage were determined after 30 and 90 days of ensiling. Additionally, the bacterial community compositions were analyzed. The *L. plantarum* inoculants significantly promoted lactic acid accumulation, and *Lactobacillus* abundance for both periods. At 90 days, silage in CK exhibited a high pH, a loss in dry matter, and a high concentration of ammoniacal nitrogen. The inoculations of *L. plantarum* significantly inhibited the growth of Clostridia, and reduced ammoniacal nitrogen concentration in silage (*P* < 0.05). Thus, inoculation with *L. plantarum* improved the fermentation quality of alfalfa silage and inhibited the growth of spoilage microorganisms, and further delayed spoilage of alfalfa silage under adverse ensiling conditions.

## Introduction

Ensiling is an anaerobic microbial-based fermentation process, dominated by lactic acid bacteria (LAB), which produce the lactic acid (LA) required for pH decline and inhibition of harmful microorganisms. It has long been a common method for forage preservation (Eikmeyer et al., 2013). Alfalfa, a widely cultivated and economically valuable pasture plant, is an important forage crop used for ensiling worldwide (Dunière et al., 2013). However, it can be hard to ensile owing to its high buffering capacity (BC) and lack of water soluble carbohydrates (WSCs) (Nkosi et al., 2016), especially when the moisture concentration exceeds 70%, resulting in clostridial fermentation (Coblentz and Muck, 2012). Wilting to a dry matter (DM) of 300–400 g/kg fresh weight (FW) is recommended before ensiling for wet grasses and legumes to prevent effluent production according to Dunière et al. (2013). However, high precipitation in some areas can make it difficult to wilt the forage before ensiling. Alfalfa is a perennial herb that is harvested in spring, summer, and autumn in most areas in China other than the north. At the end of spring or early summer, mid-eastern and southern China have abundant rainwater, and the alfalfa ensiled for over 2 or 3 months will reach high temperatures during summer. Sudden rainfall and high air humidity may cause extended wilting periods, which affect characteristics of the ensiling material. Sustained high temperatures during the ensiling process may also affect the stability of fermentation.

The microbial community plays an important role in the fermentation process and is likely to be impacted by multiple factors, such as ensiling conditions, inoculants, and epiphytic microorganisms of the fresh forage (Kasmaei et al., 2017; Guan et al., 2018). The development of PCR-based techniques enables us to define the microbial communities more accurately, and a larger variation in the microbiota in alfalfa silages than in cereal silages has been documented (McAllister et al., 2018). Inoculations of *Lactobacillus plantarum* could enhance the acidification of silage and adaption to a low pH environment, which contributes to the inhibition of competing microorganisms and effectively improves silage quality. Thus, *L. plantarum* is the most commonly used bacterial inoculant in forage ensiling studies (Oliveira et al., 2017). During ensiling, *L. plantarum* enhances LA fermentation and survives and adapts to the silage environment (Guo et al., 2018; Ogunade et al., 2018; Yang et al., 2019). However, the fermentation quality of the silage varies owing to different factors, such as BC, water activity, and presence of epiphytic microbes. The latter can differ owing to several factors, such as forage type, climatic conditions, and irrigation level. Adverse ensiling conditions, such as extremely high temperature, high humidity, and soil incorporation, can result in the poor fermentation of alfalfa silage. Under good conditions, silage without *L. plantarum* inoculation can produce a good fermentation quality, with a decline in pH, abundant LA accumulation, and sufficient LAB counts. However, few studies have examined the effects of *L. plantarum* on alfalfa silage fermentation quality when poor fermentation conditions occur (Yang et al., 2020).

The aim of this study was to investigate the mechanism of *L. plantarum* in improving fermentation quality of naturally ensiled alfalfa under the poor conditions that commonly occur in spring and summer ensiling in China. Yellow River-irrigated alfalfa was harvested during the high precipitation season when the relative humidity exceeded 80%, and ensiled for 90 days after a 1-day wilting with or without *L. plantarum* inoculation. Particular attention was paid to bacterial communities, fermentation properties, and their interactions in silage.

## Materials and Methods

### Materials and Silage Preparation

Fresh alfalfa was harvested at the early bloom stage in Zhengzhou, Henan Province (temperate monsoon climate, 34.76°N, 113.65°E, altitude 110.4 m above sea level), and wilted for 24 h. The wilted material (DM 266 g/kg FW) was chopped into 1–2-cm lengths. For inoculant preparation, *L. plantarum* YX was isolated from the Yaxin alfalfa ensiling additive (Yaxin Biotechnology Co., Ltd., Taiwan, China) and *L. plantarum* A345, an alfalfa epiphytic strain, was isolated from Shanxi, China. The *L. plantarum* strains were cultured in MRS medium at 30°C for 12 h. Then, the culture was centrifuged at 12,000 *g* for 10 min at 4°C. The precipitate was mixed with distilled water to an OD_600_ of 0.8.

Approximately 500 g for each of three replicates of the chopped alfalfa were treated with the following: (1) distilled water control (CK); (2) 1 × 10^6^ colony forming units (cfu)/g of *L. plantarum* YX (LPI); and (3) 1 × 10^6^ cfu/g of *L. plantarum* A345 (LPII). All replicates were packed into polyethylene plastic bags, vacuumed, sealed with a vacuum sealer (P-290, Shineye, Dongguan, China), and ensiled at ambient temperature (24–40°C) for 30 and 90 days.

### Analysis of Fermentation Products

The AOAC (1990) was used for DM determination. Subsamples were dried in an oven at 65°C for 48 h and pulverized to pass through a 1-mm screen using a laboratory knife mill (FW100, Taisite Instrument Co., Ltd., Tianjin, China). The WSCs were measured using anthrone colorimetry (Murphy, 1958).

Other subsamples of silage (10 g) were diluted with 90 ml of distilled water and filtered through a 0.45-μm membrane. The pH was measured using a pH meter. The organic acid contents were determined using high-performance liquid chromatography (Waters Alliance e2695, Waters, MA, United States). Carbomix H-NP 10:8% (7.8 mm × 300 mm × 10 μm) was used as the stationary phase, and the column temperature was maintained at 55°C. The injected sample volume was 10 μl. The mobile phase composition was 0.0254% sulfuric acid. The mobile phase was filtered through a 0.45-μm pore size, 47-mm diameter nylon membrane and degassed before use. The flow rate of the mobile phase was 0.6 ml/min. The detection wavelength for samples was 214 nm using a UV detector. The ammoniacal nitrogen (NH_3_-N) level was determined using Berthelot colorimetry (Broderick and Kang, 1980). The BC was determined using the hydrochloric acid–sodium hydroxide method (Playne and Mcdonald, 1996). The fermentation coefficient of alfalfa silage was predicted using the formula of Addah et al. (2011).

### Bacterial Community Analyses

Each of the 21 samples (3 bags of fresh alfalfa and 18 bags of treatments) (10 g) was mixed with 100 mL of sterile phosphate buffer saline by vigorous shaking at 180 r/min for 2 h. The mixture was filtered and the liquor was then centrifuged at 10,000 r/min for 10 min at 4°C. The deposit was resuspended in 1 mL of sterile phosphate buffer saline. The liquor was centrifuged at 12,000 r/min for 10 min at 4°C to collect microbial pellet. Total DNAs were extracted using a Bacterial DNA Kit D3350-02 (Omega Biotek, Norcross, GA, United States). The purity levels and concentrations of DNAs were evaluated by 1% agarose gel electrophoresis. The PCR amplifications of the V3–V4 regions of the bacterial 16SrDNA gene were performed using Primer F (Illumina adapter sequence 1+ CCTACGGGNGGCWGCAG) and Primer R (Illumina adapter sequence 2+ GACTACHVGGGTATCTAATCC). The PCR products were extracted from a 2% agarose gel. The amplicon sequencing of 16SrDNA was conducted using the Miseq platform (Genesky Bio-Tech Co., Ltd., Shanghai, China) after the purification and quantification of the PCR products. All the raw reads were checked using FLASH2, and low quality sequences (quality scores below 20) were discarded according to the QIIME quality control process (version 1.7.0). Operational taxonomic units were clustered using Uparse (Uparse v7.0.1001^1^). The analysis of taxonomy assignment of representative sequences was performed using the Ribosome Database Project (Cole et al., 2009). The sequence data reported in this study had been deposited in the GSA database (Accession No. CRA002694).

### Statistical Analyses

The statistical analysis of the fermentation products was performed using IBM SPSS version 21.0 (SPSS Inc., Chicago, IL, United States). The effects of different treatments were evaluated by one-way analysis of variance, with Duncan’s multiple range tests. The alpha diversity of the bacterial communities was calculated using mothur (version 1.9.1). The beta diversity analyses and correlation analyses between bacterial compositions and environmental factors were performed using R software (version 2.15.3).

## Results

### Characteristics of Wilted Alfalfa Before Ensiling

The wilted alfalfa had pH of 6.06, DM of 266 g/kg FW, WSC concentration of 17.56 g/kg DM, and a high BC of 460 mEq/kg DM. Organic acid and NH_3_-N were not detected.

### Effects of *L. plantarum* on Fermentation Properties of Alfalfa Silage

Organic acid and NH_3_-N contents, as well as the pH level, of alfalfa silage inoculated with or without *L. plantarum* strains are shown in Table 1. The *L. plantarum* inoculants effectively accelerated the LA fermentation and acetic acid (AA) accumulation, resulting in a lower pH compared with the CK group (*P* < 0.001) at 30 days; and the enhanced LA accumulation was significantly greater in the LPII group (*P* < 0.001). For 90-d silage, increase in pH level and NH_3_-N concentration, reduction in LA and WSC concentration, as well as formation of propionic acid (PA), occurred compared with 30-d silage. Significantly higher pH (*P* < 0.001) and NH_3_-N accumulation (*P* < 0.001), as well as a significant loss in DM (*P* = 0.017) were apparent in the CK compared with the inoculated groups. Silage in the LPII group had better fermentation quality compared with other groups, indicated by a relatively low pH, high LA and AA concentrations, and inhibition of NH_3_-N formation.

**TABLE 1:** Fermentation end-products after different *Lactobacillus plantarum* treatments during alfalfa ensiling.

Time	Inoculant^1^	pH	DM	LA	AA	LA/AA	PA	NH_3_-N	WSC
30 days	CK	6.10^a^	27.06	55.02^c^	40.38^c^	1.39^a^	ND	31.22^b^	6.47
	LPI	5.60^b^	26.21	66.59^b^	52.11^b^	1.28^b^	ND	35.12^a^	6.3
	LPII	5.50^b^	26.60	76.82^a^	55.70^a^	1.38^a^	ND	23.28^c^	6.94
	*P*	*P* < 0.001	0.362	*P* < 0.001	*P* < 0.001	0.034	–	*P* < 0.001	0.221
	SEM	0.05	0.39	1.78	0.74	0.02	–	0.39	0.31
90 days	CK	7.05^a^	24.72^b^	25.00^c^	48.66^ab^	0.52^c^	34.87^a^	57.06^a^	4.30^a^
	LPI	5.98^b^	26.01^a^	31.49^b^	45.31^b^	0.71^b^	25.12^b^	42.25^b^	4.83^a^
	LPII	5.67^c^	26.54^a^	42.13^a^	50.35^a^	0.84^a^	20.00^c^	36.71^b^	3.11^b^
	*P*	*P* < 0.001	0.017	*P* < 0.001	0.100	0.001	0.001	*P* < 0.001	0.007
	SEM	0.04	0.00	0.39	0.82	0.02	0.63	0.71	0.22

*Means of three observations. Values with different letters in a row indicate significant differences among treatments (*P* < 0.05). DM, dry matter; LA, lactic acid; AA, acetic acid; LA/AA, lactic acid/acetic acid; PA, propionic acid; NH_3_-N, ammoniacal nitrogen; WSC, water soluble carbohydrate. % fresh weight for DM; % total nitrogen for NH_3_-N; g/kg of DM for LA, AA, PA, and WSC. ^1^CK, control; LPI, inoculated with *L. plantarum* YX; LPII, inoculated with *L. plantarum* A345. SEM, standard error of means.*

### Effects of *L. plantarum* on Bacterial Community Composition

**FIGURE 1:**
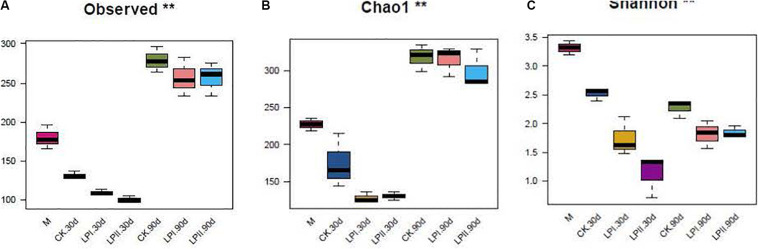
Box-plots of observed bacterial species **(A)**, Chao 1 **(B)** and Shannon indices **(C)** of bacterial communities in alfalfa silage. M, alfalfa material before ensiling; CK, control; LPI, inoculated with *L. plantarum* YX; LPII, inoculated with *L. plantarum* A345. The numbers following CK, LPI, and LPII stand for ensiled days of silage. Observed, observed bacterial species. *P < 0.05; **P < 0.01.

High-throughput analysis was used to determine the bacterial diversity in alfalfa silage, and the valid sequences were clustered into 552 operational taxonomic units based on a 97% sequence identity. Richness and diversity indices of the bacterial communities, represented by observed species, the Chao1 and the Shannon indexes, respectively, are shown in Figures 1A–C. The richness of the bacterial community decreased after ensiling for 30 days compared with the fresh material, and then increased at 90 days (*P* = 0.005). The diversity of the bacterial community decreased after ensiling (*P* = 0.006). The diversity of the bacterial community in silage in LPII slightly and non-significantly increased at 90 days compared with 30-d silage (*P* = 0.1).

**FIGURE 2:**
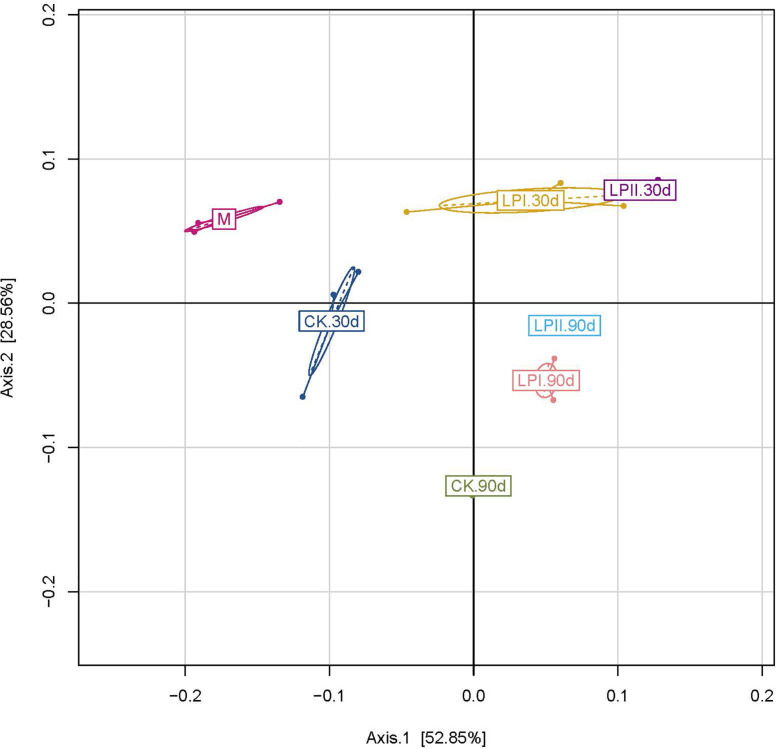
Cluster analysis of bacterial communities in alfalfa silage as assessed by a Principal Coordinate Analysis. M, alfalfa material before ensiling; CK, control; LPI, inoculated with *L. plantarum* YX; LPII, inoculated with *L. plantarum* A345. The numbers following CK, LPI, and LPII stand for ensiled days of silage.

The principal coordinate analysis (PCoA) clearly reflected the variation within the bacterial community (Figure 2). The clear separation between bacterial communities of the wilted alfalfa and alfalfa silage indicated a shift after ensiling. Divisions in the plots representing silage with and without inoculants for both periods indicated that the distribution of the bacterial community was shifted by *L. plantarum* inoculations. The distribution of the bacterial communities among the three replications within LPII was more similar compared to those within CK and LPI at 30 days. Although the Shannon indexes were similar between 30-d and 90-d silage (Figure 1C), divisions in the plots representing silage for the two periods indicated a variation within the bacterial community.

**FIGURE 3:**
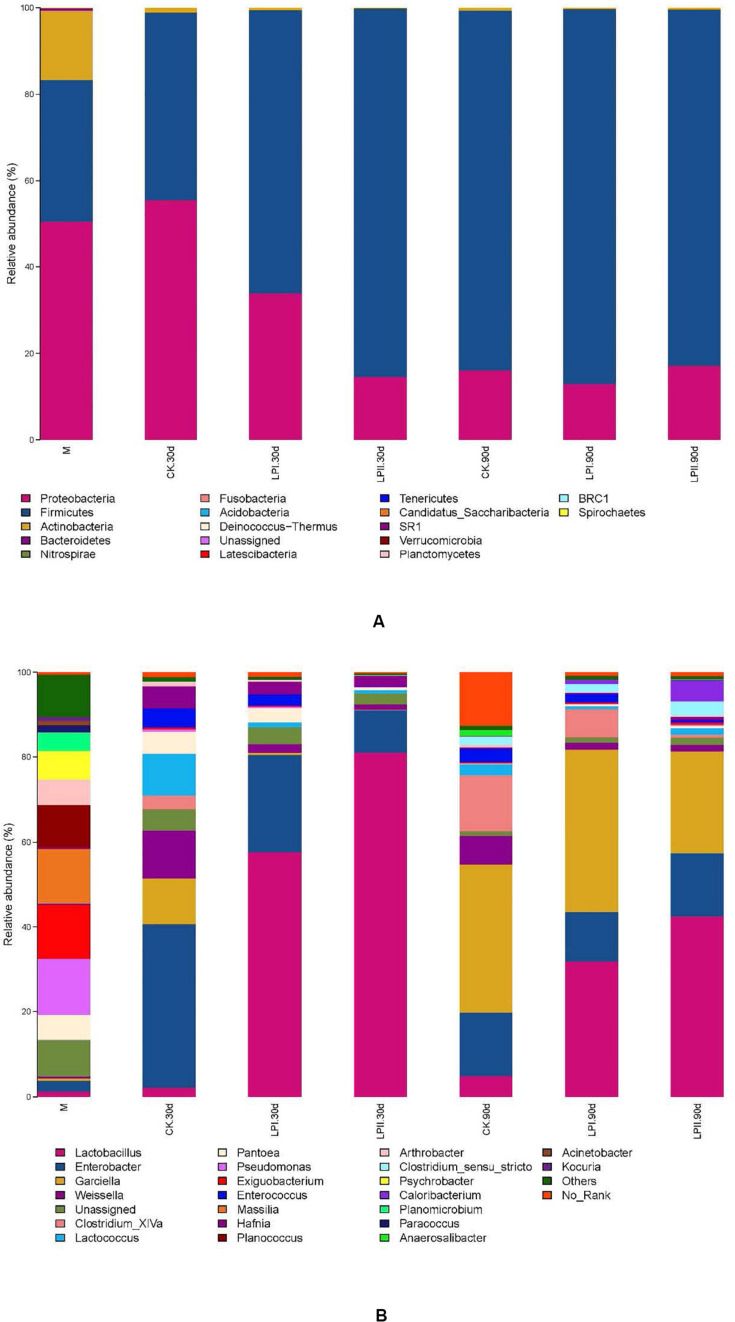
Bacterial community and relative abundances by phylum **(A)** and genus **(B)** for alfalfa silage. M, alfalfa material before ensiling; CK, control; LPI, inoculated with *L. plantarum* YX; LPII, inoculated with *L. plantarum* A345. The numbers following CK, LPI, and LPII stand for ensiled days of silage.

Proteobacteria (52.14%) was the predominant phylum in the wilted alfalfa, followed by Firmicutes (30.33%) (Figure 3A). Firmicutes became the predominant phylum after ensiling, except for the CK group at 30 days. Inoculation increased the relative abundance of Firmicutes in the 30-d silage compared with CK, and the relative abundance of Firmicutes was greater in LPII (84.74%) than LPI (64.62%). At the genus level, *Pseudomonas* (15.35%), *Exiguobacterium* (12.36%), *Massilla* (14.15%), and *Planococcus* (8.00%) dominated the epiphytic bacterial community of alfalfa, while abundance levels of these genera reduced after ensiling. Low abundance of LAB species was exhibited in the alfalfa epiphytic bacterial community, including *Lactobacillus* (1.36%), *Weissella* (0.70%), *Lactococcus* (0.07%), *Pediococcus* (0.01%), and *Leuconostoc* (<0.01%). A complex bacterial community composition was exhibited in CK in the 30-d silage, with the prevalent genera being *Enterobacter* (38.54%), *Weissella* (11.59%), *Garciella* (10.42%), and *Lactococcus* (9.59%) (Figure 3B). However, *Lactobacillus*, the predominant genus in LPI and LPII (57.82 and 82.19%, respectively), only exhibited a relative abundance of 2.05% in CK at 30 days. Inoculation inhibited the growth of *Enterobacter* at 30 days, with the lowest relative abundance for LPII (10.20%). The relative abundance of *Enterobacter* became similar among the three treatments in the 90-d silage (14.96% in CK, 11.60% in LPI, and 14.83% in LPII). *Garciella* became a prevalent genus at 90 days, with relative abundance of 34.95% in CK, 38.38% in LPI, and 24.04% in LPII. The relative abundance of *Lactobacillus* in the inoculated silage decreased, and Clostridia including *Clostridium_XlVa* and *Clostridium_sensu_stricto* grew in all treatments at 90 days compared with 30-d silage. An inhibitory effect against *Clostridium_XlVa* was apparent in LPII compared with the other two groups in 90-d silage.

**FIGURE 4:**
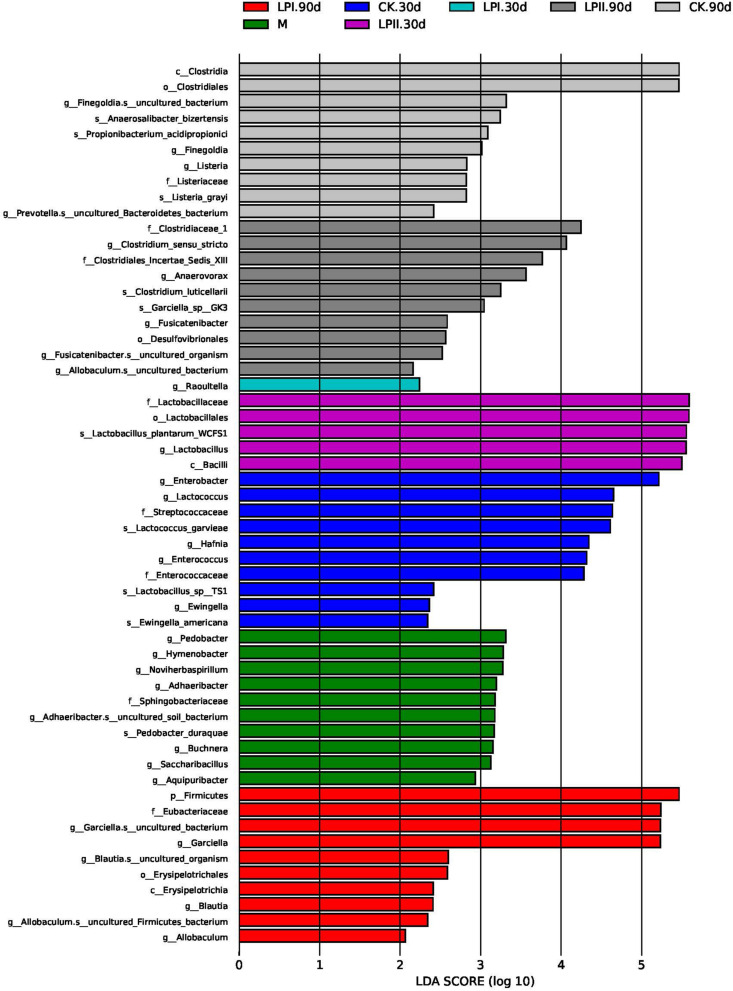
Comparison of microbial variations using LefSe analysis. M, alfalfa material before ensiling; CK, control; LPI, inoculated with *L. plantarum* YX; LPII, inoculated with *L. plantarum* A345. The numbers following CK, LPI, and LPII stand for ensiled days of silage.

LefSe was performed to further explore the variations in the bacterial communities among the treatments (Figure 4). Significantly higher abundances of species of *Enterobacter*, *Lactococcus*, and *Enterococcus* were observed in CK at 30 days. *Lactobacillus* species grew well in LPII in 30-d silage. At 90 days, the relative abundance of some species belonging to spoilage genus *Clostridium* and pathogenic genus *Listeria* were significantly higher in the CK group. Although silage in LPII effectively inhibited the growth of *Clostridium_XlVa* (Figure 3B) (13.42% in CK, 6.67% in LPI, and 0.67% in LPII, respectively), it showed a relatively poorer inhibition against some Clostridia genera with low abundance. *Clostridium_sensu_stricto*, for instance, a genus belonging to family Clostridiaceae_1, exhibited relative abundances of 1.94, 1.88, and 3.23% in CK, LPI, and LPII in 90-d silage, respectively.

### Correlation Analysis of the Bacterial Community With Fermentation Products

Mantel tests were performed to reveal the relationships between bacterial community compositions at different taxon levels and silage properties (Table 2). Results indicated significant correlations between fermentation properties and the bacterial community composition (*P* < 0.05) at different taxon levels, except for pH value and bacterial community composition at phylum level. Spearman’s correlation analysis indicated that the pH value and LA concentration had significant correlations with richness and diversity indices of the bacterial communities (*P* < 0.05). A strong negative correlation was found between AA concentration and the Shannon index (*P* < 0.05). Meanwhile, the number of observed species and the Chao1 index of the bacterial community showed positive correlations with PA and NH_3_-N concentrations, and negative correlations with WSC concentration (*P* < 0.05).

**TABLE 2:** Correlation of bacterial community with fermentation properties in alfalfa silage illustrated by Mantel test and Spearman’s correlation analysis.

	pH		LA		AA		PA		NH_3_-N		WSC	
	r	*P*	r	*P*	r	*P*	r	*P*	r	*P*	r	*P*
**Mantel test**												
Phylum	0.070	0.141	0.737	0.000	0.188	0.020	0.784	0.000	0.173	0.028	0.525	0.001
Class	0.163	0.033	0.759	0.000	0.254	0.003	0.772	0.000	0.215	0.013	0.503	0.001
Order	0.174	0.024	0.760	0.000	0.245	0.004	0.777	0.000	0.216	0.010	0.501	0.001
Family	0.301	0.003	0.750	0.000	0.350	0.001	0.706	0.000	0.265	0.005	0.501	0.000
Genus	0.295	0.002	0.748	0.000	0.352	0.000	0.720	0.000	0.261	0.005	0.504	0.000
**Spearman’s correlation analysis**												
Observed^1^	0.706	0.001	−0.928	0.000	−0.178	0.481	0.887	0.000	0.726	0.001	−0.731	0.001
Chao1	0.649	0.004	−0.897	0.000	−0.172	0.494	0.872	0.000	0.598	0.009	−0.637	0.004
Shannon	0.740	0.000	−0.484	0.042	−0.635	0.005	0.228	0.362	0.406	0.095	−0.407	0.094

*LA, lactic acid; AA, acetic acid; PA, propionic acid; NH_3_-N, ammoniacal nitrogen; WSC, water soluble carbohydrate. ^1^Observed, observed bacterial species.*

**FIGURE 5:**
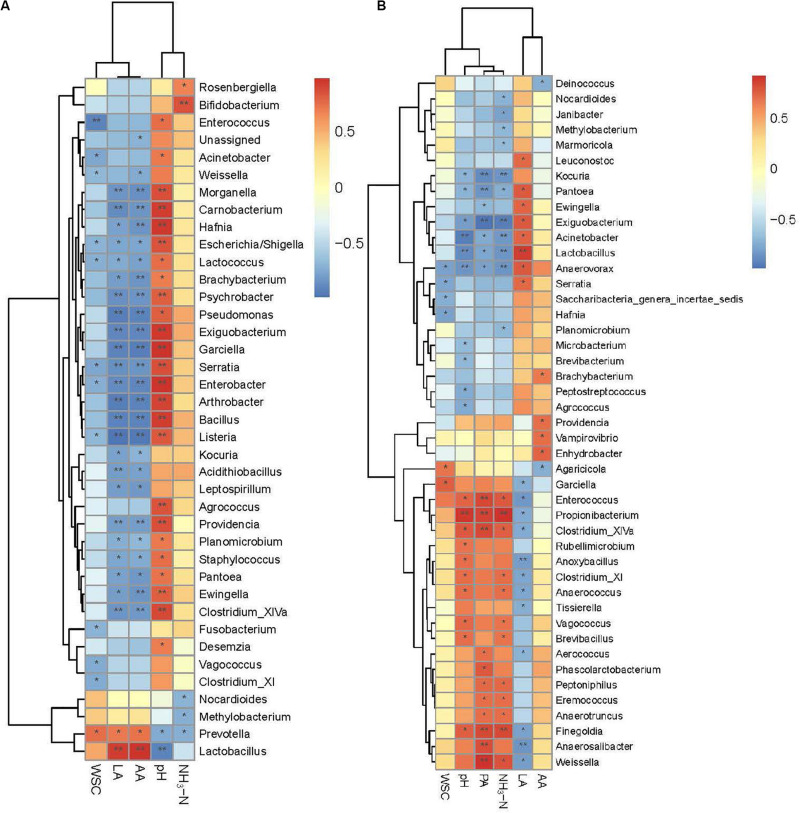
Spearman correlation heatmap of abundance of bacterial genera and fermentation properties in alfalfa ensiled for 30 days **(A)** and 90 days **(B)**. LA, lactic acid; AA, acetic acid; LA/AA, lactic acid/acetic acid; PA, propionic acid; NH_3_-N, ammoniacal nitrogen; WSC, water soluble carbohydrate. *P < 0.05; **P < 0.01.

Spearman’s correlations further illustrated the relationships between bacterial genera and silage properties for different periods (Figures 5A,B). *Lactobacillus* was positively correlated with LA (*r* = 0.92 at 30 days and 0.87 at 90 days), and negatively correlated with pH (*r* = −0.92 at 30 days and −0.88 at 90 days) for both periods. Positive correlations were found between NH_3_-N and two genera at 30 days (*P* < 0.05), *Rosenbergiella* and *Bifidobacterium*. The NH_3_-N was negatively correlated with *Nocardioides*, *Methylobacterium*, and *Prevotella* in 30-d silage (*P* < 0.05). The NH_3_-N was positively correlated with a series of nitrogen-fermenting genera at 90 days, including *Anaerotruncus*, *Peptoniphilus*, and genera belonging to Clostridia (*Clostridium_XlVa*, *Clostridium_XI*, and *Finegoldia*) (*P* < 0.05). Other genera also showed positive correlations with NH_3_-N at 90 days, including *Weissella*, *Eremococcus*, *Brevibacillus*, *Vagococcus*, *Anaerococcus*, *Enterococcus*, and *Propionibacterium* (*P* < 0.05). *Nocardioides* and *Methylobacterium* retained their negative correlations with NH_3_-N at 90 days. Another genus belonging to Nocardioidaceae, *Marmoricola*, was also negatively correlated with NH_3_-N. Negative correlations with NH_3_-N were also apparent for the acid-producing genus *Lactobacillus*, nitrate-reducing genera *Pantoea* and *Exiguobacterium*, putrescine-fermenting genus *Anaerovorax*, and other genera, including *Planomicrobium*, *Acinetobacter*, *Kocuria*, and *Janibacter*, with unknown functions in the ensiling process.

## Discussion

### Characteristics of Wilted Alfalfa

Adverse conditions during harvesting and ensiling could result in spoilage of silage. In this study, Yellow River-irrigated alfalfa was chosen as the ensiling material, and was harvested at the early bloom stage during the high precipitation season. Although the wilting period was extended compared with previous studies (Li X. et al., 2018; Li J. et al., 2018), the DM (266 g/kg FW) was still lower than the recommended content (300–400 g/kg FW) (Yuan et al., 2017). This might due to the high precipitation during the harvest season. Frequent rainfall resulting in high air humidity slowed the moisture loss during wilting. As reported by Agarussi et al. (2019) and Tao et al. (2017), wilting decreased the WSC concentration and crude protein content in fresh alfalfa. The 24-h wilting may have exacerbated the lack of WSC (17.56 g/kg DM) in this study. The WSC of the wilted alfalfa was lower than 50 g/kg DM, which was insufficient for adequate fermentation during ensiling (Ni et al., 2018). The BC was still high after wilting in this study. Frequent rainfall might increase the incorporation of soil in fresh alfalfa during harvest, resulting in an increase in BC (Weinberg and Ashbell, 2003). This might stimulate the epiphytic aerobic microorganisms to remain active for an extended period and so reduce the quantity of WSCs for further LAB fermentation. The low fermentation coefficient (26.9 < 35) also suggested that the material was too hard to ensile naturally (Ke et al., 2015).

### Effects of *L. plantarum* on Fermentation Properties of Alfalfa Silage

Inoculation with *L. plantarum* had a positive effect on fermentation properties in terms of lower pH and higher LA accumulation. Similar effects were also reported by Wang et al. (2019) and Yang et al. (2019), in which LA fermentation was accelerated by *L. plantarum* inoculants in *Moringa oleifera* leaves and in alfalfa silage, respectively. The LPII exhibited enhanced LA fermentation than LPI. This might be explained by better adaption of the alfalfa epiphytic inoculant in LPII. Good adaption to the alfalfa ensiling environment of the inoculant aids in improvement of silage quality (Zhao et al., 2020). The AA concentrations increased with *L. plantarum* inoculations at 30 days. The AA is a promoter of aerobic stability during the ensiling process (Schmidt and Kung, 2010) and an effective inhibitor of fungi (Le Lay et al., 2016). This enhancement in AA accumulation might help with reduction in microbial richness at early stages of ensiling. Although inoculation enhanced LA fermentation in the 30-d silage, pH in the silage was highly above ideal level (<4.20) (Wang et al., 2017). The increase in pH in 90-d silage also suggested that LA fermentation was insufficient to stabilize the ensiled mass. The insufficient LA fermentation might be due to a lack of available substrate and high BC of the wilted alfalfa.

The LA to AA ratio became much lower at 90 days in all treatments, indicating reduction in LA fermentation in the silage. The PA contents in silage were also out of the acceptable range of 1–10 g/kg DM (Agarussi et al., 2019). The NH_3_-N is considered a representative of amino acid deamination and decarboxylation (Scherer et al., 2015), which generally reduce the nutritional value of silage. The remarkable increase in pH value and NH_3_-N concentration, as well as the loss in DM in CK at 90 days indicate potential spoilage of silage. The delay in spoilage with *L. plantarum* inoculation was shown by the lower pH and NH_3_-N concentration, higher LA residual, and good DM maintenance. The NH_3_-N concentration in LPII was significantly lower compared with other treatments, indicating better protein maintenance with the inoculant.

Notably, the NH_3_-N concentration increased in LPI (*P* < 0.001), but the LA accumulation and pH decline were greater, compared with the CK group. Effects of Clostridia and plant proteolytic enzymes may be typical causes of NH_3_-N accumulation (Kung and Shaver, 2001). Tao et al. (2012) reported that most plant proteolytic enzymes in alfalfa silage showed greater activities at pH 5.0–6.0, which could explain the higher NH_3_-N concentration in LPI. Plant proteolytic enzymes tended to be more active at the pH level in LPI (5.60) than in CK (6.10). Although LPI and LPII had similar pH values (5.60 and 5.50, respectively), LPII showed low NH_3_-N formation. This might result from the greater acidogenic capability of the inoculant in LPII in terms of higher LA and AA concentrations, which contributed to an increased inhibition of proteolytic microorganisms.

### Effects of *L. plantarum* on Bacterial Community of Alfalfa Silage

Although richness of bacterial community decreased compared with the wilted alfalfa at the early stage of ensiling, the richness flourished at 90 days. Similar trends were also reported by Zheng et al. (2017) in direct-cut alfalfa silage with and without LAB inoculant and sugar, in which richness of the bacterial community decreased at the early stage of ensiling compared with the alfalfa material, and later increased during the ensiling process. Consistent with the study of Ni K. et al. (2017), diversity of the bacterial community decreased after ensiling. Inoculation slightly reduced diversity of the bacterial community. Although the Shannon indexes were similar between the 30-d and 90-d silage, the PCoA showed variation within the bacterial community. The clear separation between bacterial communities of the wilted alfalfa and alfalfa silage indicated a shift after ensiling, consistent with previous studies (Ni K. K. et al., 2017; Yang et al., 2019). Divisions between the plots representing silage with and without inoculants indicated that the distribution of the bacterial community was shifted by *L. plantarum* inoculations, which could explain the differences in fermentation quality (Ni et al., 2018). The distribution of the bacterial communities among the three replications within LPII was more similar compared with those within LPI for both periods. The more stable bacterial community distribution in LPII might result from its stronger LA fermentation represented by the highest LA concentration (Table 1) and *Lactobacillus* abundance (Figure 3B) among the treatments for both periods.

Proteobacteria (52.14%) was the most predominant phylum in the wilted alfalfa, while abundance of Proteobacteria and Firmicutes reversed after being ensiled for 90 days, with Firmicutes dominating the bacterial community. Keshri et al. (2018) concluded that environmental conditions that developed during ensiling favor the selection of species of the phylum Firmicutes that flourish under low pH and anaerobic conditions. The increasing abundance of Firmicutes at 30 days in inoculated silage might indicate an acceleration of the LA fermentation process in the presence of *L. plantarum*. Acceleration of the LA fermentation with *L. plantarum* inoculation was also shown at the genus level. The major bacteria involved in LA fermentation of silage belong to the genera *Lactobacillus*, *Pediococcus*, *Weissella*, and *Leuconostoc* (Pang et al., 2011; Ni et al., 2018). Low abundance of these genera was exhibited in the wilted alfalfa before ensiling, and aerobic microorganisms dominated the bacterial community. This was consistent with the results of Tao et al. (2017), in which a long period of wilting decreased the LAB count and promoted growth of aerobes in alfalfa before ensiling.

A series of Clostridia genera grew in the 90-d silage. Clostridia may lead to excessive protein degradation, DM loss, and butyric acid (BA) production in silage, and further promote the growth of other less acid-tolerant spoilage microorganisms (Wang et al., 2019). Thus, they are undesirable in silage. Clostridiales (64.47%) dominated the bacterial community in CK at 90 days at the order level. Inoculation with *L. plantarum* effectively inhibited the dominance of Clostridiales (48.27% in LPI and 29.26% in LPII, respectively), which further reduced NH_3_-N formation, maintained the DM (Table 1), and inhibited less acid-tolerant *Listeria* pathogens in silage (Figure 4). The increased Clostridiales abundance and pH values in alfalfa silages indicated a secondary fermentation at 90 days (Flythe and Russell, 2004), which led to LA consumption, and accumulation of some weaker volatile fatty acids like AA, PA, and BA. This was consisted with the presence of PA in 90-d silage (Table 1). Although Clostridia is a major producer of BA in silage, presence of BA was not detected in our study. This might result from the activity of BA-utilizing microbes in silage. For instance, relative abundance of *Aerococcus*, a butyrate-producing saccharolytic genus, increased at 90 days in all the three treatments compared with 30-d silage (*P* < 0.05). Similarly, relative abundance of *Serratia*, whose presence is typically associated with 2,3-butanediol production (McDonald et al., 1991), increased at 90 days in LPII (0.03% at 30 days, 0.11% at 90 days). Flythe and Russell (2004) reported that some Clostridia could produce large amounts of AA apart from BA. This might explain the high AA concentration in CK at 90 days. A high abundance of *Garciella*, an anaerobic and thermophilic bacterium belonging to Clostridiales (Miranda-Tello et al., 2003), was apparent in the 90-d silage. A high abundance level of *Garciella* was also reported by Zhang et al. (2018) in alfalfa silage ensiled at a high temperature of 40°C. The relatively high temperature during July (with the highest temperature over 40°C during the day time) might be one cause of *Garciella* growth. Spearman’s correlation analysis showed a weak relationship between *Garciella* and NH_3_-N (Figure 5B). In all, the current evidence is insufficient to indicate a certain role of *Garciella* in the ensiling process.

Although *L. plantarum* inoculation promoted *Lactobacillus* abundance in silage, it failed to stabilize the abundance of *Lactobacillus* at 90 days. The overlong period of wilting before ensiling may have exacerbated the poor stability of the bacterial community. The period of wilting reduced the WSC concentration available for LA fermentation, and led to a lower LAB population before ensiling. Occurrence of continuing relatively high temperature at daytime during summer (38–40°C) may also exacerbated the hardness for LAB dominance under high moisture condition. With the pH values in silage highly above ideal level (4.2), such environmental condition is suitable for the growth of some harmful microorganisms like *Enterobacter* and *Clostridium*, which competes with LAB growth.

### Relationships Between Bacterial Community and Fermentation Properties

Recent studies illustrated an interaction of bacterial community and silage properties (McAllister et al., 2018). Mantel tests revealed close correlations of the pH, LA, AA, PA, NH_3_-N, and WSC with bacterial community composition. The pH and LA affected both the richness and diversity of bacterial community. The concentration of AA remained stable for both periods (Table 1) regardless of the shift in bacterial abundance. This is consistent with its poor relationship with bacterial richness illustrated by Spearman correlations. There was a negative correlation between AA and diversity of the bacterial community, possibly due to an inhibitory effect of AA toward some bacterial species. The PA and NH_3_-N had positive correlations with bacterial richness, but this did not affect diversity of the bacterial community. A probable explanation is that PA and NH_3_-N were mainly produced by some specific species in silage, so concentrations of these products mainly depended on the number of their producers rather than the composition of microbes. The WSC was negatively correlated with bacterial abundance. This is predictable, because WSC is consumed with the growth of microbes, as a main nutrient source of microbes in silage.

Spearman’s correlation analysis illustrated some potential spoilage genera in alfalfa silage. Genera that correlated with NH_3_-N differed between the two periods. This indicates a potential shift in the main promoters of NH_3_-N formation during ensiling. At 30 days, *Rosenbergiella* and *Bifidobacterium* were potential promoters of NH_3_-N formation. *Rosenbergiella* is a genus belonging to Enterobacteriaceae. An abundance of *Bifidobacterium* was also reported in direct-cut alfalfa silage with a high NH_3_-N concentration (Zheng et al., 2017). At 90 days, presence of Clostridia lead to increasing formation of NH_3_-N by fermenting amino acids (Flythe and Russell, 2004). Spearman’s correlation analysis illustrates a series of NH_3_-N producers belonging to Clostridia, including *Clostridium_XlVa*, *Clostridium_XI*, and *Finegoldia*. Another two nitrogen-utilizing genera, *Anaerotruncus* and *Peptoniphilus*, were also considered to promote NH_3_-N formation. There is little evidence concerning the role of *Eremococcus* in silage fermentation. One recent study reported dominance of *Eremococcus* in the anodic biofilms in air–cathode microbial fuel cells (Zhang et al., 2016). In air–cathode microbial fuel cells, electroactive microorganisms, mainly in the form of anodic biofilms, accelerate the rate of electrochemical oxidation of complex organic substrates (Borole et al., 2011). This indicates the potential capability of *Eremococcus* in oxidizing organic materials; however, this study did not show that oxidation by *Eremococcus* could occur under acidic conditions. Thus, further research is needed to explore its role in the ensiling system. *Anaerococcus* is a butyrate-producing saccharolytic genus, and so may exhibit synergy with *Clostridium* during ensiling – this might explain its positive correlation with NH_3_-N. Some species of *Vagococcus* were reported to exhibit capability for arginine hydrolysis (Teixeira et al., 1997), which might aggravate protein degradation in silage. In this study, *Weissella* was outcompeted by *Lactobacillus* following *L. plantarum* inoculation, and exhibited low abundance in the inoculated groups. This might explain the positive correlation between *Weissella* and NH_3_-N. A higher abundance of *Weissella* was exhibited in CK, for which the NH_3_-N concentration was significantly higher than the other treatments. *Propionibacterium* requires complex nutrition, including multiple vitamins and an abundant nitrogen source to produce PA and AA from glucose and other carbohydrates (Rogers et al., 2006). Accumulation of NH_3_-N might promote its growth and fermentation metabolism, resulting in the high PA concentrations in the 90-d silage. *Brevibacillus* was also positively correlated with NH_3_-N, but its role in ensiling is currently unclear.

The *L. plantarum* inoculation induced a rapid drop in pH at 30 days, which would inhibit proteolytic bacteria and contribute to true protein preservation (Weinberg and Muck, 1996). This was also illustrated by the inhibitory effect against Clostridiales in the inoculated silage and the negative correlation between *Lactobacillus* and NH_3_-N. Apart from *Lactobacillus*, correlation analysis suggests that some other genera may also contribute to protein preservation. However, the roles of these genera in the ensiling process remain little understood, and more evidence is needed to elucidate their relationships with protein preservation in silage.

## Conclusion

In conclusion, high precipitation in spring and summer during harvest and high temperatures during the ensiling process of alfalfa are universal in mid-eastern and southern China, as well as other areas with similar climatic characteristics. Inoculations of *L. plantarum* significantly promoted pH decline, LA accumulation, and *Lactobacillus* abundance under such poor fermentation conditions in alfalfa silage. Growth of Clostridia were inhibited after *L. plantarum* inoculations, which further reduced NH_3_-N formation in the 90-d silage. Thus, *L. plantarum* improved the fermentation properties of alfalfa silage under adverse ensiling conditions by enhancing LA fermentation and inhibiting the growth of spoilage microorganisms, and further delayed the spoilage of silage.

## Author’s Note

This manuscript has been released as a pre-print at ResearchSquare, Applied & Industrial Microbiology.

## Data Availability Statement

The datasets presented in this study can be found in online repositories. The names of the repository/repositories and accession number(s) can be found in the article/supplementary material.

## Author Contributions

FY: conceptualization, formal analysis, writing – original draft, and visualization. YaW: writing – review and editing, supervision, project administration, and funding acquisition. SZ: methodology, validation and investigation. YuW: resources. All authors contributed to the article and approved the submitted version.

## Conflict of Interest

The authors declare that the research was conducted in the absence of any commercial or financial relationships that could be construed as a potential conflict of interest.

## Footnotes

1 http://www.drive5.com/uparse/

## Abbreviations

AA: acetic acid
BA: butyric acid
BC: buffering capacity
DM: dry matter
FW: fresh weight
LA: lactic acid
LAB: lactic acid bacteria
LDA: linear discriminant analysis
NH_3_-N: ammoniacal nitrogen
PA: propionic acid
WSC: water soluble carbohydrate.
